# Liquiritigenin Ameliorates Rheumatoid Arthritis by Modulating the Nrf2/NF-κB/NLRP3 Pathway in Fibroblast-like Synoviocytes

**DOI:** 10.3390/ph19050785

**Published:** 2026-05-17

**Authors:** Zhuoxi Chen, Nana Chen, Limin Liu, Yingrui Wang, Lejian Zhu, Hui Yang, Zhuqi Han, Xiaoyu Zhang, Shuo Yan, Yuan Du, Leiming Zhang

**Affiliations:** 1 School of Traditional Chinese Medicine, Shandong Medical and Pharmaceutical University, Yantai 264003, China; 2 Key Laboratory of Molecular Pharmacology and Drug Evaluation (Yantai University), Ministry of Education, School of Pharmacy, Yantai University, Yantai 264005, China

**Keywords:** liquiritigenin, rheumatoid arthritis, fibroblast-like synoviocytes, ROS, inflammation, Nrf2/NF-κB/NLRP3 pathway

## Abstract

**Background/Objectives:** Rheumatoid arthritis (RA) is an autoimmune disorder manifesting as joint destruction and synovial inflammation, with the aberrant activation of fibroblast-like synoviocytes (FLSs) functioning as a critical pathological mechanism. Liquiritigenin (LIQ), a natural flavonoid extracted from licorice root, possesses anti-inflammatory and antioxidant activities; however, its efficacy and mechanism in RA pathological models remain unclear. This study aimed to investigate the anti-RA effects of LIQ mediated through FLSs and its underlying mechanisms. **Methods:** Complete Freund’s adjuvant (CFA)-induced rat model and TNF-α-stimulated MH7A cell model were employed to assess the anti-RA effects and underlying mechanisms. In vivo experiments examined the effects of LIQ on RA manifestations, joint damage, and inflammatory responses in CFA-induced rats, while in vitro experiments explored its effects on aberrant activation, oxidative stress, and inflammation in TNF-α-stimulated MH7A cells. The regulatory effects of LIQ on the Nrf2/NF-κB/NLRP3 signaling pathway were validated by immunofluorescence and Western blotting in vivo and in vitro. **Results:** LIQ alleviated joint swelling and bone damage, reducing synovial cellular infiltration and hyperplastic changes in CFA-induced rats. Furthermore, LIQ inhibited proliferation, migration, and invasion while reducing reactive oxygen species levels in TNF-α-stimulated MH7A cells, and decreased IL-1β and IL-18 levels in rat serum and MH7A cell supernatants. Moreover, LIQ activated Nrf2 and inhibited NF-κB and NLRP3, thereby attenuating inflammatory responses and alleviating oxidative stress. Administration of the Nrf2 inhibitor ML385 partially reversed its suppressive effects on inflammatory responses and oxidative stress in vivo and in vitro. **Conclusions:** LIQ exerted anti-RA effects in FLSs by suppressing inflammation and aberrant activation. Its mechanism may involve modulation of the Nrf2/NF-κB/NLRP3 signaling pathway.

## 1. Introduction

Rheumatoid arthritis (RA) is a persistent autoimmune condition that primarily affects joint tissues, presenting clinically with joint pain, ongoing inflammation, and difficulties in movement [1]. The prevalence of RA worldwide varies between 0.1% and 1.6%, exhibiting notable regional variations and a higher occurrence in females [2,3]. Fibroblast-like synoviocytes (FLSs) serve as a critical contributor to the pathological process of RA. Under chronic inflammatory stimulation, FLSs in the joint exhibit aberrant activation, manifesting as excessive proliferation, migration, and invasion, which collectively leads to synovial hyperplasia, destruction of cartilage and bone structures, and secretion of pro-inflammatory mediators that amplify inflammation [4,5]. Targeting the abnormally activated FLSs is considered a key therapeutic target for RA progression.

The signaling pathway of nuclear factor-κB (NF-κB) functions as a critical transcriptional regulator orchestrating inflammatory responses throughout RA progression [6]. Upon stimulation by pathogen-associated molecular patterns (PAMPs), inflammatory cytokines or damage-associated molecular patterns (DAMPs), IκB-α undergoes phosphorylation followed by ubiquitin-proteasome degradation, enabling the NF-κB heterodimer (p50/p65) to undergo nuclear translocation and activate pro-inflammatory gene expression, thereby amplifying synovial inflammation and promoting bone destruction [7]. The NOD-like receptor protein 3 (NLRP3) inflammasome pathway is another key pro-inflammatory signaling pathway mediated by NF-κB in RA progression [8]. The NLRP3 inflammasome is composed of NLRP3, apoptosis-associated speck-like protein containing a CARD (ASC), and caspase-1. When activated, nuclear NF-κB promotes the expression of NLRP3 assembly proteins and precursors of interleukin (IL)-1β and IL-18, providing substrates for inflammasome formation and function and regulating the activation process by modifying the structure of the NLRP3 protein [9,10]. When activated by signals like reactive oxygen species (ROS), potassium efflux or adenosine triphosphate, the NLRP3 inflammasome processes cytokine precursors and releases these mature inflammatory mediators into the extracellular environment, causing a vicious positive-feedback cycle of synovial inflammation in RA [8]. As such, suppressing both NF-κB and NLRP3 pathways represents a promising way to treat RA.

As an oxidative stress factor, ROS have been identified as an important driving force that escalates synovial inflammation in RA. In the RA joint microenvironment, ROS induce IκB-α degradation, thereby activating the NF-κB pathway and exerting pro-inflammatory effects. Concurrently, ROS drive inflammasome substrate polymerization, thereby enabling NLRP3-dependent secretion of pro-inflammatory mediators [11,12]. The nuclear factor erythroid 2-related factor 2/heme oxygenase-1 (Nrf2/HO-1) pathway represents a classical intracellular antioxidant defense system. Its activation rapidly lowers ROS levels and restrains NF-κB nuclear translocation and NLRP3 inflammasome initiation, generating a dual “antioxidant-anti-inflammatory” shield [13]. Thus, in RA, this pathway is regarded as a critical route for suppressing oxidative damage and inflammatory activation of FLSs.

Traditional treatments for RA mainly consist of disease-modifying antirheumatic drugs, nonsteroidal anti-inflammatory drugs and glucocorticoids. Although these drugs can effectively reduce symptoms of RA, they may also be linked to various side effects. Methotrexate (MTX) stands as the preferred initial therapeutic option for RA pharmacotherapeutic management, often utilized in single-agent protocols or combined with complementary medications [14]. Despite its significant role in RA management, the side effects of MTX limit its long-term safety, including gastrointestinal reactions, liver damage, and bone marrow suppression [15]. In contemporary research, the application of natural plant-derived compounds in RA treatment has garnered widespread attention. Various natural compounds have shown substantial anti-inflammatory and immune-modulating potential in RA models, or when combined with conventional agents such as MTX to improve therapeutic efficacy and attenuate side effects [16,17,18]. These studies suggest that natural plant components hold broad application prospects in RA treatment, potentially offering safer and more effective therapeutic options for patients.

**Figure 1:**
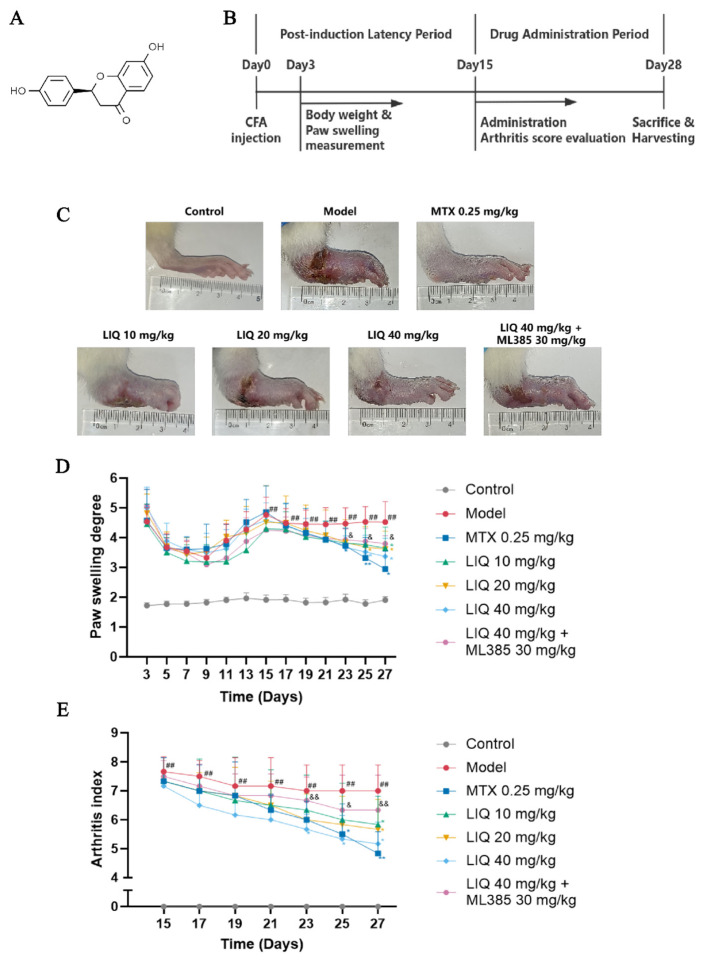
LIQ attenuates RA progression in CFA-induced rats. (**A**) Chemical structure of LIQ. (**B**) Schematic diagram of the experimental protocol for the CFA-induced rat model. (**C**) Images of the hind paws of rats. (**D**,**E**) Determination of (**D**) paw-swelling degree and (**E**) arthritis index. All data were presented as the mean ± SD (*n* = 6–8). ^##^ *p* < 0.01 vs. control group; * *p* < 0.05, and ** *p* < 0.01 vs. model group; and ^&^ *p* < 0.05 and ^&&^ *p* < 0.01 vs. ML385 group.

Licorice (*Glycyrrhiza glabra* L.) is acknowledged as one of the most ancient and extensively utilized medicinal plants in the world, having served as a therapeutic agent in various medical traditions [19]. According to previous literature, licorice extract and its bioactive constituents can alleviate pathological joint damage in animal models of RA [20]. Liquiritigenin (LIQ) (Figure 1A), a naturally occurring flavanone isolated from licorice roots, exhibits multiple bioactivities encompassing anti-inflammatory, antioxidant, antibacterial and hepatoprotective effects [21]. Its anti-inflammatory and protective actions have been documented in models of liver injury, renal injury and colitis [22,23,24]. However, the potential therapeutic effects of LIQ on RA and its molecular mechanisms remain to be fully elucidated. Accordingly, the current research sought to investigate the efficacy of LIQ in a complete Freund’s adjuvant (CFA)-induced rat model and in a tumor necrosis factor-α (TNF-α)-induced MH7A cell model, thereby elucidating the molecular mechanisms underlying its anti-RA properties.

## 2. Results

### 2.1. Effect of LIQ on RA Symptoms in CFA-Induced Rats

The experimental timeline for CFA-induced RA model establishment and drug administration was shown in Figure 1B. Rats receiving CFA model treatment developed arthritis symptoms, manifesting as polyarticular inflammation including swelling, erythema, joint stiffness and deformity (Figure 1C). Paw swelling in CFA-treated rats showed a slight decrease followed by an increase from Day 0 to Day 15, with both paw swelling and arthritis index peaking at Day 15. During the administration period, treatment with LIQ and MTX alleviated RA symptoms in CFA-induced rats and decreased paw swelling and arthritis index, with these effects becoming particularly pronounced after Day 23. Compared with LIQ alone, ML385 partially reversed the therapeutic effect of LIQ, as evidenced by increased paw swelling and arthritis index (Figure 1D,E).

### 2.2. Effect of LIQ on Joint Damage and Inflammatory Responses in CFA-Induced Rats

**Figure 2:**
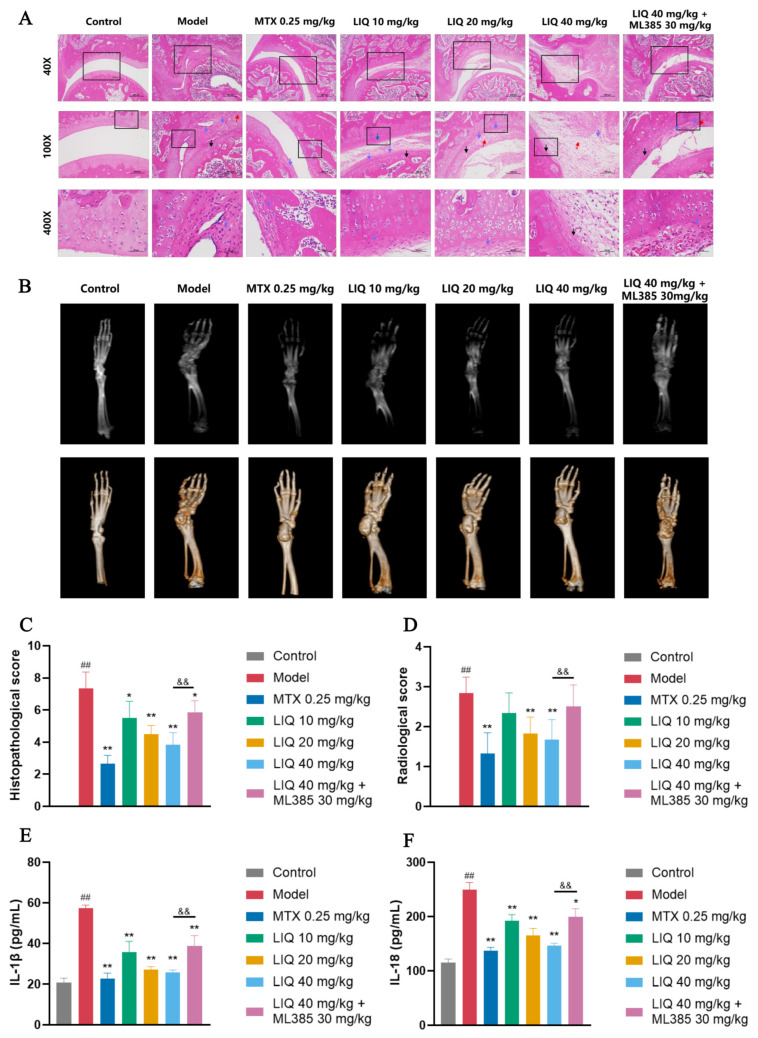
LIQ alleviates joint damage and suppresses inflammation in CFA-induced rats. (**A**) Histopathological images of joint tissues by HE staining in CFA-induced rats. Arrows indicate typical lesions: red, inflammatory cell infiltration; blue, synovial hyperplasia; black, cartilage erosion. Original magnification, 40×; high-power views, 100× and 400×. (**B**) Radiological images via micro-CT and corresponding 3D reconstruction of joint tissues in CFA-induced rats. (**C**,**D**) The evaluation of (**C**) histopathological scores and (**D**) radiological scores of joint tissues. (**E**,**F**) Levels of inflammatory cytokines (**E**) IL-1β and (**F**) IL-18 in rat serum. All data were presented as the mean ± SD (*n* = 4). ^##^ *p* < 0.01 vs. control group; * *p* < 0.05 and ** *p* < 0.01 vs. model group; and ^&&^ *p* < 0.01 vs. ML385 group.

In the ankle joint hematoxylin and eosin (HE)-stained sections of normal rats in the control group, the synovial lining was thin and intact, with no obvious inflammatory cell infiltration, a smooth cartilage surface, and continuous cortical bone. CFA-induced rats exhibited marked thickening of the synovial lining, dense inflammatory cell infiltration, and extensive cartilage erosion relative to controls. Treatment with LIQ and MTX alleviated these inflammatory and destructive lesions and reduced the histopathological scores. The protective benefits of LIQ were attenuated by ML385, leading to aggravated inflammatory synovial hyperplasia and erosive joint damage (Figure 2A,C).

Micro computed tomography (micro-CT) scanning and 3D reconstruction were employed to analyze joint and bone damage in rats. The control group exhibited smooth, intact bones with clearly defined joint structures. The model group exhibited severe joint destruction predominantly affecting the ankle, tarsal, and metatarsophalangeal joints. These joints presented with markedly irregular bony contours, loss of smooth outlines, and diffuse erosion. The ankle joints showed the most pronounced changes, leading to significantly elevated radiological scores. Treatment with LIQ and MTX significantly ameliorated ankle joint damage, reduced the extent of affected joints and bone, and lowered the radiological scores. ML385 partially reversed the protective effects of LIQ on joint and bone damage (Figure 2B,D).

The concentrations of IL-1β and IL-18 in the serum of rats were measured through enzyme-linked immunosorbent assay (ELISA) among different treatment groups. The group treated with CFA showed markedly increased concentrations of IL-1β and IL-18. Conversely, the treatment with either LIQ or MTX resulted in a significant reduction in the concentrations of both IL-1β and IL-18. In contrast to LIQ on its own, ML385 abrogated the inhibitory effects of LIQ on IL-1β and IL-18 levels. (Figure 2E,F).

### 2.3. Effects of LIQ on the Immune Organ Index and Body Weight in CFA-Induced Rats

**Figure 3:**
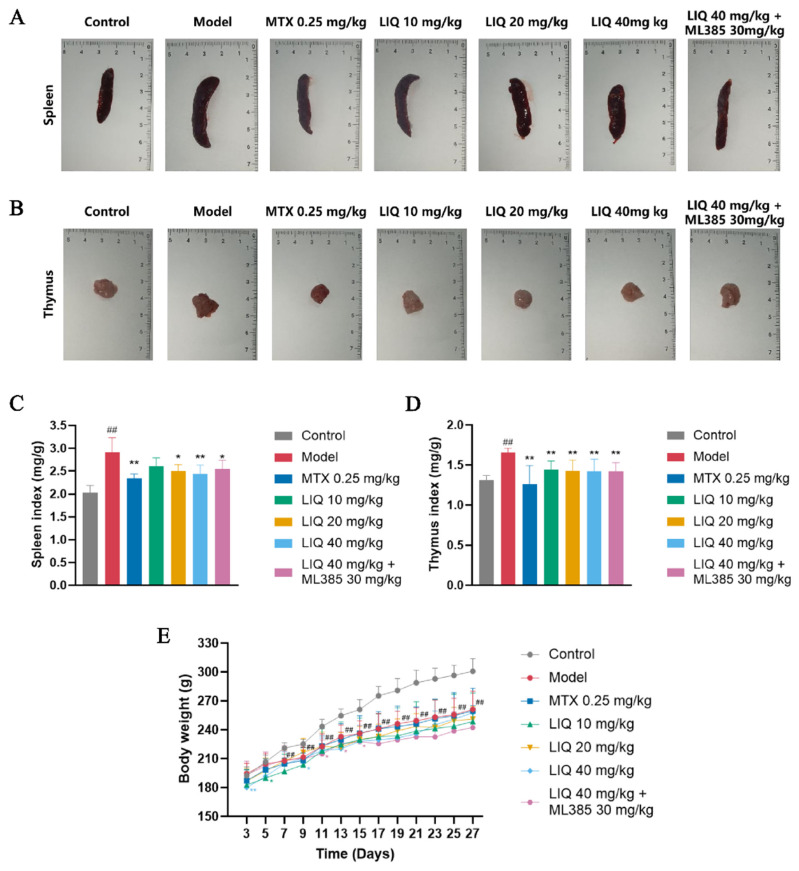
LIQ reduces immune organ indices without significant effects on body weight in CFA-induced rats. (**A**,**B**) Images of (**A**) spleen and (**B**) thymus in rats. (**C**,**D**) Measurement and evaluation of (**C**) spleen index and (**D**) thymus index in rats. (**E**) Body weight in rats throughout the experimental period. All data were presented as the mean ± SD (*n* = 4 for **A**–**D**; *n* = 6–8 for **E**). ^##^ *p* < 0.01 vs. control group; * *p* < 0.05 and ** *p* < 0.01 vs. model group.

Representative images of the spleen and thymus from each group are shown in Figure 3A,B. CFA-induced rats exhibited substantial elevations in both spleen and thymus indices relative to the control group. Treatment with LIQ or MTX markedly reduced spleen and thymus indices in CFA-induced rats. However, ML385 exerted no significant effect on either the spleen or thymus indices of LIQ-treated rats (Figure 3C,D). Body weight measurements showed that all rats experienced a consistent rise in body weight during the experimental period; however, the body weights of the CFA-treated groups were lower compared with those of the control group; meanwhile, drug administration did not significantly alter body weight compared to the model group (Figure 3E).

### 2.4. Effects of LIQ on the Nrf2/NF-κB/NLRP3 Pathway in CFA-Induced Rats

**Figure 4:**
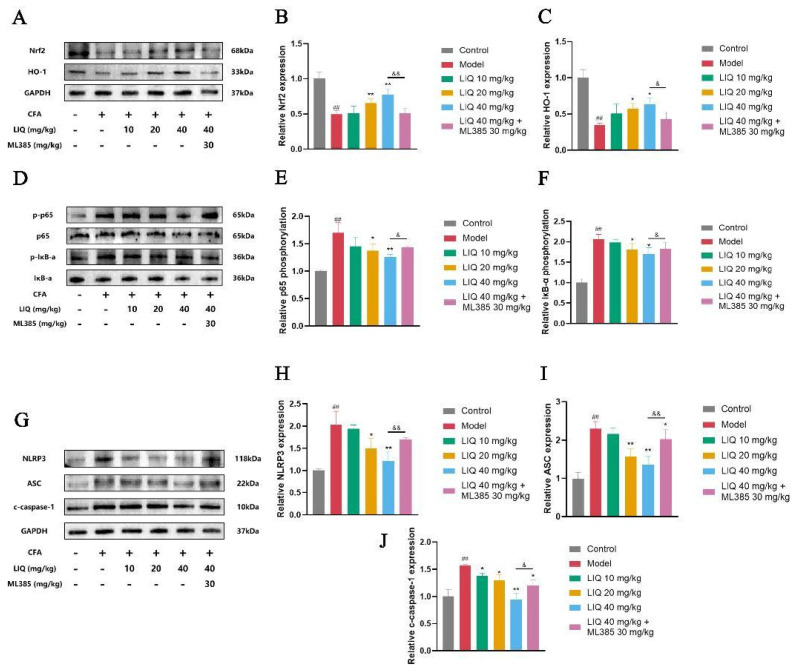
LIQ regulates the Nrf2/NF-κB/NLRP3 pathway in CFA-induced rats. Western blotting was employed to assess the effects of LIQ on the Nrf2/NF-κB/NLRP3 pathway. (**A**–**C**) The expression levels of (**B**) Nrf2 and (**C**) HO-1. (**D**–**F**) The phosphorylation levels of (**E**) p65 and (**F**) IκB-α. (**G**–**J**) The expression levels of (**H**) NLRP3, (**I**) ASC and (**J**) c-caspase-1. All data were presented as the mean ± SD of three independent experiments. ^##^ *p* < 0.01 vs. control group; * *p* < 0.05 and ** *p* < 0.01 vs. model group; and ^&^ *p* < 0.05 and ^&&^ *p* < 0.01 vs. ML385 group.

The Western blot analysis was performed to investigate the effects of LIQ on Nrf2, NF-κB and NLRP3 inflammasome in rat joint tissues. CFA treatment markedly reduced Nrf2 and HO-1 expression. In contrast, administering LIQ enhanced the expression of both Nrf2 and HO-1. As an inhibitor of Nrf2, ML385 significantly attenuated the upregulating effect of LIQ on Nrf2 and HO-1 in rat joint tissues (Figure 4A–C).

NF-κB signaling serves as a pivotal modulator of inflammatory processes in RA. The Western blot analysis further revealed that CFA administration significantly enhanced the activation of NF-κB in joint tissues, as demonstrated by the elevated phosphorylation levels of p65 and IκB-α. LIQ treatment, in contrast, significantly reduced the phosphorylation levels of both p65 and IκB-α. ML385 attenuated LIQ-mediated suppression of NF-κB, restoring the elevated phosphorylation of p65 and IκB-α (Figure 4D–F).

The NLRP3 inflammasome pathway mediates inflammation progression and release of IL-1β and IL-18 in RA. In the Western blot analysis, CFA administration elevated the expression of NLRP3, ASC, and cleaved (c)-caspase-1. Relative to the CFA model group, LIQ treatment reduced the levels of all three proteins. Conversely, ML385 restored the expression of NLRP3, ASC, and c-caspase-1 in LIQ-treated joint tissues (Figure 4G–J).

### 2.5. Effect of LIQ on Aberrant Activation and Inflammatory Cytokine Secretion in TNF-α-Induced MH7A Cells

**Figure 5:**
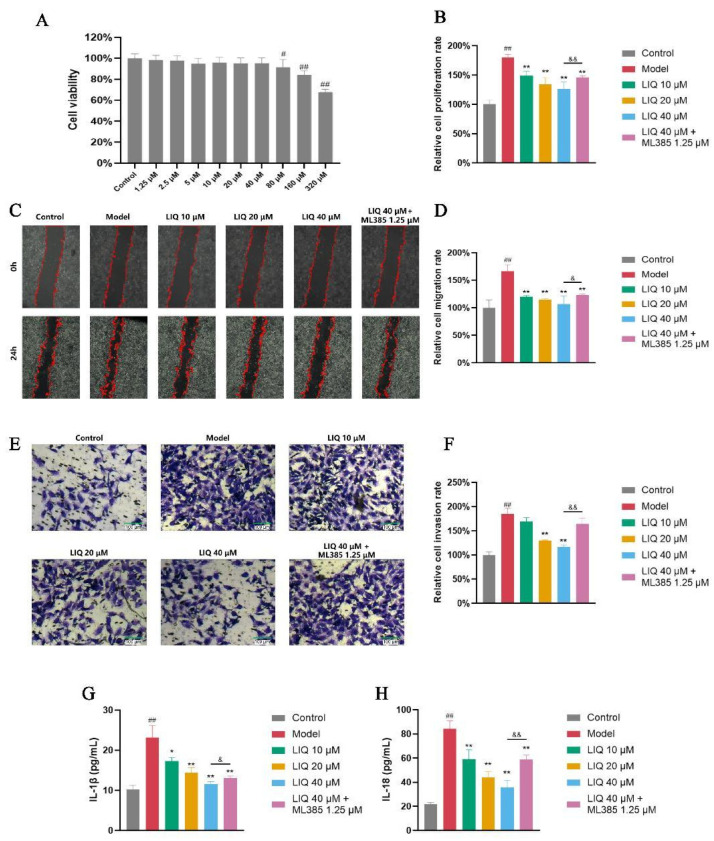
LIQ suppresses aberrant activation and release of inflammatory cytokines in TNF-α-induced MH7A cells. (**A**) Effects of LIQ on MH7A cell viability detected by MTT assay to determine the dose range. (**B**) Cell proliferation evaluated by MTT assays. (**C**,**D**) Cell migration evaluated by scratch wound healing assays. (**E**,**F**) Cell invasion evaluated by Transwell assays. (**G**,**H**) Levels of inflammatory cytokines (**G**) IL-1β and (**H**) IL-18 in supernatants of MH7A cells. All data were presented as the mean ± SD of three independent experiments. ^##^ *p* < 0.01 vs. control group; * *p* < 0.05 and ** *p* < 0.01 vs. model group; and ^&^ *p* < 0.05 and ^&&^ *p* < 0.01 vs. ML385 group.

LIQ at concentrations up to 40 μM had no significant effect on MH7A cell viability (Figure 5A). Proliferation assays via methyl thiazolyl tetrazolium bromide (MTT) were employed to investigate the effects of LIQ on the proliferation of TNF-α-stimulated MH7A cells. Treatment with the inflammatory cytokine TNF-α significantly enhanced the proliferative capacity of MH7A cells. LIQ effectively inhibited the proliferative activity of TNF-α-stimulated MH7A cells. Meanwhile, ML385 partially countered the suppressive effect of LIQ on the proliferation of MH7A cells (Figure 5B).

Proliferation, migration and invasion represent important manifestations of the aberrant FLS activation in the pathological process of RA. Wound healing and Transwell assays showed that 24 h stimulation with TNF-α markedly increased the migratory and invasive capacities of MH7A cells, as evidenced by increased scratch wound healing areas (Figure 5C,D) and the quantity of cells moving to the lower chamber of the Transwell inserts (Figure 5E,F). Compared with the TNF-α-treated group, LIQ dose-dependently reduced the scratch wound healing area and decreased the number of migrated cells. Compared with LIQ alone, ML385 significantly attenuated the reduction in wound closure and the number of invading cells.

The secretion of cytokines is a pivotal process driving cellular inflammation responses. TNF-α stimulation significantly elevated the concentrations of the cytokines IL-1β and IL-18 in supernatants of MH7A cells. Treatment with LIQ dose-dependently reduced the secretion of IL-1β and IL-18. ML385 attenuated the inhibitory effects of LIQ on IL-1β and IL-18 (Figure 5G,H).

### 2.6. Effects of LIQ on the Levels of ROS and the Nrf2 Expression in TNF-α-Induced MH7A Cells

**Figure 6:**
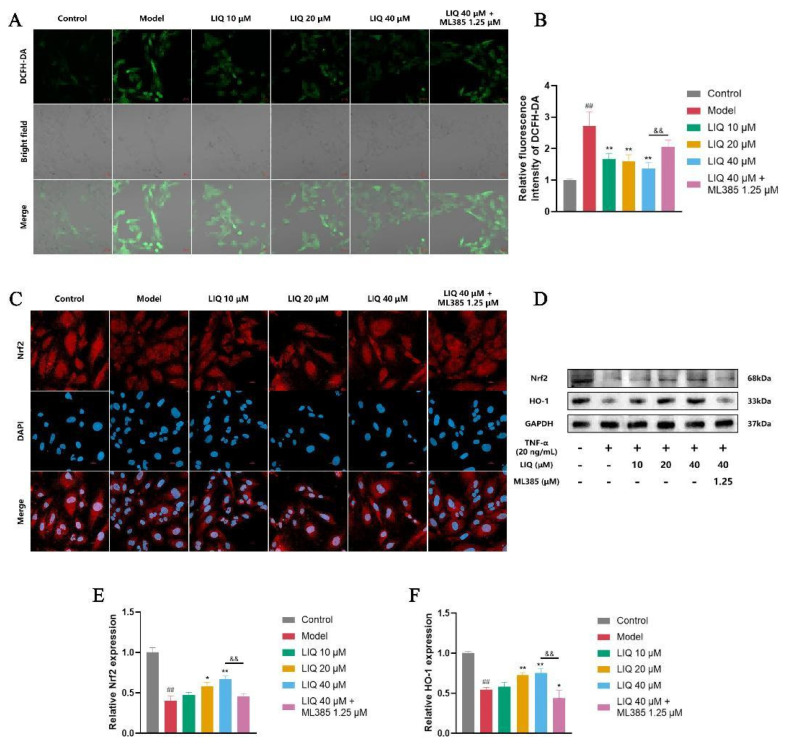
LIQ reduces ROS levels and upregulates the expression of Nrf2 and HO-1 in TNF-α-induced MH7A cells. Fluorescence probe staining, immunofluorescence staining and Western blotting were employed to evaluate the effects of LIQ on Nrf2. (**A**,**B**) ROS levels were measured by the DCFH-DA probe (green) with corresponding brightfield images showing cell morphology and localization. Scale bar = 20 μm. (**C**) The expression levels of Nrf2 via immunofluorescence staining. Scale bar = 20 μm. (**D**–**F**) The expression levels of (**E**) Nrf2 and (**F**) HO-1 via Western blotting. All data were presented as the mean ± SD of three independent experiments. ^##^ *p* < 0.01 vs. control group; * *p* < 0.05 and ** *p* < 0.01 vs. model group; and ^&&^ *p* < 0.01 vs. ML385 group.

ROS are involved in the promotion of inflammatory damage and induction of oxidative damage, with their levels under regulatory control of the Nrf2 pathway. The fluorescence method involving 2′,7′-dichlorofluorescin diacetate (DCFH-DA) was employed to measure the levels of ROS in MH7A cells. Following TNF-α treatment, there was a notable increase in ROS levels in MH7A cells. LIQ dose-dependently reduced the ROS levels in TNF-α-stimulated MH7A cells. ML385 partially reversed the inhibitory effects of LIQ, leading to a rebound in ROS levels (Figure 6A,B).

The Nrf2 pathway regulates cellular antioxidant defense, and its effect on Nrf2 expression in MH7A cells was assessed using immunofluorescence and Western blotting. As shown in Figure 6C, immunofluorescence revealed that TNF-α stimulation decreased Nrf2 expression. LIQ restored Nrf2 expression in TNF-α-stimulated MH7A cells, an effect that was partially abrogated by ML385 co-treatment. Western blot analyses corroborated these findings, showing that LIQ upregulated both Nrf2 and HO-1 in TNF-α-stimulated MH7A cells. ML385 blunted the LIQ-induced up-regulation of Nrf2 and HO-1, causing the expression of both proteins to decline once again (Figure 6D–F).

### 2.7. Effects of LIQ on the Nuclear Translocation of NF-κB and Expression of NLRP3 in TNF-α-Induced MH7A Cells

**Figure 7:**
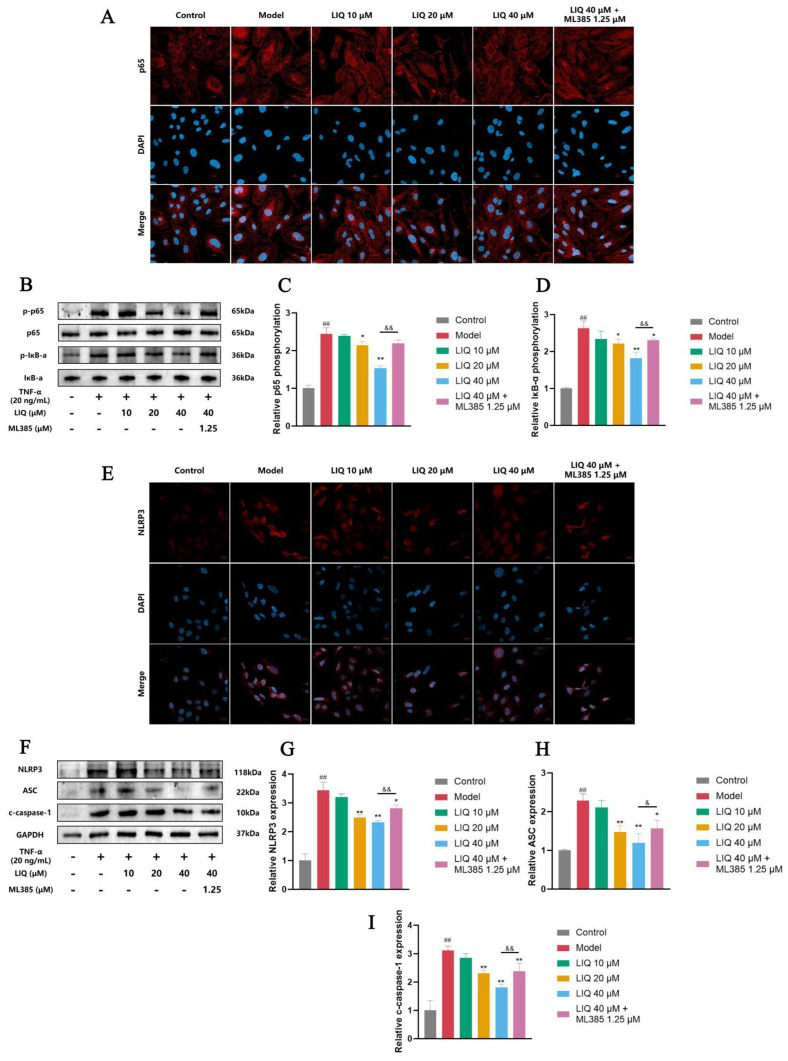
LIQ inhibits the nuclear translocation of NF-κB and the expression of the NLRP3 inflammasome in TNF-α-induced MH7A cells. (**A**) The nuclear translocation of p65 via immunofluorescence staining. Scale bar = 20 μm. (**B**–**D**) The phosphorylation levels of (**C**) p65 and (**D**) IκB-α via Western blotting. (**E**) The expression levels of NLRP3 via immunofluorescence staining. Scale bar = 20 μm. (**F**–**I**) The expression levels of (**G**) NLRP3, (**H**) ASC and (**I**) c-caspase-1. All data were presented as the mean ± SD of three independent experiments. ^##^ *p* < 0.01 vs. control group; * *p* < 0.05 and ** *p* < 0.01 vs. model group; and ^&^ *p* < 0.05 and ^&&^ *p* < 0.01 vs. ML385 group.

Activation of the NF-κB signaling pathway is primarily indicated by the nuclear translocation of the p65 subunit. Immunofluorescence revealed that LIQ effectively inhibited the nuclear translocation of NF-κB p65 in MH7A cells that were stimulated with TNF-α, whereas subsequent ML385 treatment restored p65 nuclear import (Figure 7A). Western blot analyses corroborated these findings: LIQ reduced TNF-α-stimulated phosphorylation of both p65 and IκB-α, while ML385 attenuated this inhibitory effect, re-elevating phosphorylation levels of the two proteins (Figure 7B–D). Regarding the NLRP3 inflammasome, immunofluorescence (Figure 7E) and Western blotting (Figure 7F–I) revealed that LIQ reduced the levels of NLRP3, ASC and c-caspase-1 in TNF-α-stimulated MH7A cells. Conversely, ML385 mitigated this suppressive effect, leading to a restoration of NLRP3, ASC and c-caspase-1 expression levels.

## 3. Discussion

RA is an inflammatory autoimmune disorder primarily impacting the joints, with pathological manifestations mainly including joint swelling, pain, and restricted movement [25]. Our study demonstrated that LIQ alleviated RA severity, reduced paw volume, and decreased arthritis scores in CFA-induced rats. Furthermore, synovial inflammation and bone destruction are characteristic histopathological manifestations of RA [26]. The results showed that LIQ suppressed synovial inflammatory infiltration and bone damage in the joints of CFA-induced rats as evidenced by histopathological and radiological assays. These findings indicated the potential therapeutic efficacy of LIQ against RA. Additionally, LIQ administration in CFA-induced rats maintained stable body weight trends, with no behavioral abnormalities or adverse reactions observed.

Dysregulated elevated proliferative activity and enhanced migratory and invasive capacities represent the core phenotypic hallmarks of aberrant activation in RA-FLS, constituting a critical pathogenic basis for driving joint damage in RA. Moreover, the aberrant proliferation and migration of FLSs can synergize with other pathogenic effects, such as the release of inflammatory cytokines and immune cell recruitment, collectively contributing to the characteristic synovial inflammation and structural joint destruction of RA [27]. In the in vitro study, LIQ markedly reduced the proliferation, migration, and invasion of MH7A cells stimulated by TNF-α. These results are consistent with the amelioration of arthritis symptoms and joint-protective effects observed in LIQ-treated rats, suggesting the potential of LIQ to alleviate RA through suppression of aberrant FLS activation.

Inflammatory responses are a defining characteristic of RA [28]. The spleen and thymus participate in immunomodulation through T cell production and activation, with their morphological and functional changes reflecting systemic inflammatory conditions in RA progression [29]. At the mechanistic level, IL-1β and IL-18 are pivotal cytokines that drive RA progression through inflammatory responses and bone destruction [30]. Targeted inhibition of these cytokines has proven therapeutically effective in attenuating RA severity [31,32]. In this study, administration of LIQ reduced spleen and thymus indices in CFA-induced rats, and decreased IL-1β and IL-18 levels in both rat serum and TNF-α-stimulated MH7A cell supernatants. These results indicate the anti-inflammatory and immunomodulatory effects of LIQ in RA models, as well as its suppressive action on the key pro-inflammatory cytokines IL-1β and IL-18.

The NLRP3 inflammasome pathway, acting as a predominant producer of IL-1β and IL-18, exerts a pivotal influence on RA onset and progression. Its activation requires a two-step regulation: First, the activated NF-κB promotes the expression of the assembly protein NLRP3 and the functional precursors of IL-1β and IL-18. Following this, these NLRP3-related proteins are activated by RA-related signals (such as PAMPs or DAMPs), which facilitate inflammasome assembly. The activated inflammasome leads to the generation of mature caspase-1 to process the cytokine precursors, resulting in the production of IL-1β and IL-18 [8]. The NF-κB signaling operates as a critical mediator of inflammatory responses and NLRP3 inflammasome activation. A previous study has demonstrated that suppressing NF-κB signaling can alleviate the severity of RA and ameliorate the enhanced proliferation of FLSs [33]. Our in vitro study demonstrated that LIQ downregulated the levels of NLRP3, ASC and c-caspase-1. Immunofluorescence staining and Western blotting assays revealed that LIQ blocked p65/IκB-α phosphorylation and nuclear p65 translocation. These findings support that LIQ exerts its anti-arthritic and anti-inflammatory effects in RA via blockade of the NF-κB and NLRP3 inflammasome.

**Figure 8:**
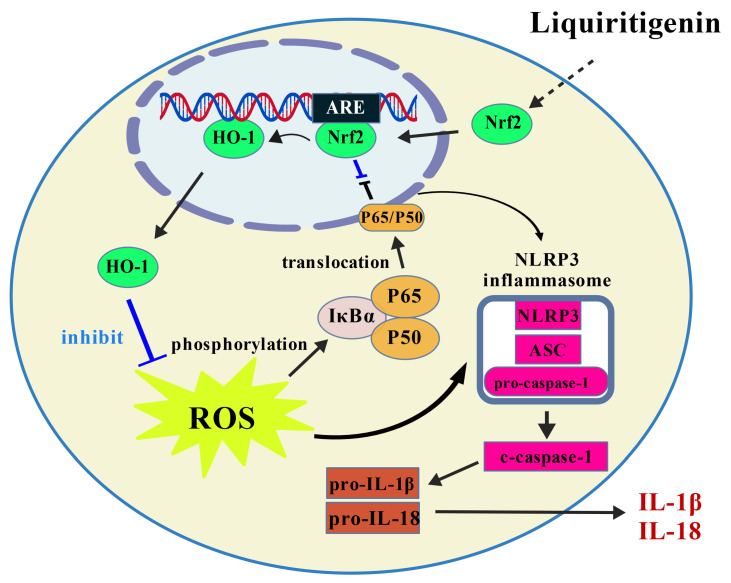
Simplified schematic diagram of the mechanism underlying LIQ’s therapeutic effects in RA models. Aberrant activation of FLSs is intimately linked to inflammation and oxidative stress. Upon inflammatory cytokine stimulation, FLSs generate excessive ROS and activate NF-κB, which collectively drives the assembly and activation of the NLRP3 inflammasome, ultimately resulting in the release of IL-1β and IL-18 that exacerbates the inflammatory joint microenvironment and accelerates RA progression. LIQ can suppress ROS generation through activation of Nrf2, thereby attenuating NF-κB and NLRP3 activation and inhibiting the secretion of IL-1β and IL-18. These effects collectively attenuate aberrant FLS activation, thereby alleviating pathological joint damage during RA progression.

ROS serve as essential intermediaries that connect oxidative stress and inflammation, significantly influencing the modulation of NF-κB signaling and the activity of the NLRP3 inflammasome [34]. Nrf2 signaling represents a critical intracellular defense system against oxidative stress. Under homeostatic conditions, Nrf2 remains confined to the cytoplasm through interaction with Kelch-like ECH-associated protein 1 (KEAP1), which facilitates its ubiquitin-dependent breakdown. In response to oxidative stress or activators, Nrf2 detaches from KEAP1, migrates into the nucleus, and associates with antioxidant response elements, consequently inducing the expression of downstream genes encoding antioxidant and detoxifying enzymes like HO-1, thereby attenuating ROS and toxic metabolites [35]. Previous studies have indicated that Nrf2 attenuates pro-inflammatory pathways including NF-κB and NLRP3 by competitively binding to gene loci or mediating the production of anti-inflammatory metabolites, thereby exerting both anti-inflammatory and antioxidant effects [13]. Our experimental results indicated that LIQ increased the expression of Nrf2 and HO-1 in CFA-induced rats and TNF-α-stimulated MH7A cells. In vivo experiments revealed that LIQ downregulated ROS levels and inhibited NF-κB and NLRP3 activation. These findings are consistent with LIQ-mediated Nrf2 activation. The application of the ML385 inhibitor further supported the central regulatory role of Nrf2 in RA. Compared with LIQ treatment alone, ML385 partially reversed the ameliorative and joint-protective effects of LIQ in CFA-induced rats, reinstated aberrant activation in TNF-α-stimulated MH7A cells, suppressed Nrf2 activation and elevated intracellular ROS levels, and upregulated expression of NF-κB and NLRP3. Collectively, the anti-RA efficacy of LIQ and its suppression of NF-κB/NLRP3 are associated with Nrf2 activation (Figure 8).

It is noteworthy that the experimental models employed in this study reflect salient pathophysiological features relevant to human RA. The CFA-induced adjuvant arthritis rat model reproduces key disease hallmarks, including synovial inflammation and joint destruction, serving as a standard preclinical platform for anti-arthritic drug screening [36,37]; concurrently, the MH7A cell line, derived from human synovial fibroblasts, retains typical phenotypic characteristics of RA-FLS, providing an in vitro system for mechanistic investigation [38]. This study integrated the regulatory effects of LIQ on the Nrf2/NF-κB/NLRP3 signaling in RA-FLS, which were validated through both in vivo and in vitro experiments, ensuring the robustness and reliability of our conclusions.

However, this study has several limitations. Mechanistically, the present study primarily focused on the Nrf2/NF-κB/NLRP3 axis, leaving the modulation of other RA-related pathways (such as MAPK or JAK/STAT) by LIQ and their potential crosstalk with this axis to be fully elucidated. Additionally, neither the CFA model nor the immortalized MH7A cell line fully recapitulates the chronic polyarticular autoimmunity and cellular heterogeneity of human RA. Future studies employing collagen-induced arthritis models and primary patient-derived FLSs will provide greater translational relevance and further elucidate the therapeutic potential and mechanisms of LIQ.

## 4. Materials and Methods

### 4.1. Reagents

LIQ (CAS: 578-86-9, formula: C_15_H_12_O_4_, purity ≥ 98%, HPLC) was obtained from Jingzhu Biotechnology (Nanjing, China). Nrf2 inhibitor ML385 (CAS: 846557-71-9, formula: C_29_H_25_N_3_O_4_S, purity ≥ 99%) was purchased from MedChemExpress (Monmouth Junction, NJ, USA). CFA was sourced from Chondrex Inc. (Redmond, WA, USA). MTX (purity ≥ 98%) and TNF-α were obtained from Solarbio (Beijing, China). ROS and MTT Assay Kits were obtained from Beyotime Biotechnology (Shanghai, China). ELISA kits for cytokines IL-1β and IL-18 were obtained from Xinyu Biotechnology (Shanghai, China). Primary antibodies targeting Nrf2, HO-1, phosphorylated (p)-NF-κB p65, NF-κB p65 and NLRP3 were obtained from Huabio (Hangzhou, China). Antibodies against p-IκB-α, IκB-α, ASC, c-caspase-1 and GAPDH were sourced from Sanying Biotechnology (Proteintech Group, Wuhan, China). Other reagents (analytical grade) were purchased from standard commercial sources.

### 4.2. Animals

The Sprague Dawley rats (male, 150–180 g) were sourced from Shandong Pengyue Experimental Animal Technology (Jinan, China; License: SCXK [LU] 2022-0006). Rats were kept in a regulated setting at 22 ± 2 °C under a 12 h: 12 h light–dark cycle, and received laboratory-grade feed and fresh water without restriction. All animals were housed in a controlled laboratory setting and given 1 week to acclimate before the experiments began.

### 4.3. Induction of Adjuvant Arthritis

The RA rat model was developed by administering 0.1 mL of CFA that contained 10 mg/mL of heat-killed Mycobacterium tuberculosis into the plantar area of the right hind paw of the rats [39]. In the control group, rats were administered an equivalent volume of saline at the identical site. The commencement of the experiment was marked by CFA injection and recorded as Day 0.

### 4.4. Group Allocation and Administration

The CFA-treated rats were randomly allocated to the model group, MTX group (0.25 mg/kg), various doses of LIQ groups (10, 20, 40 mg/kg), and ML385 group (40 mg/kg LIQ + 30 mg/kg ML385) with an initial cohort of 10 rats per group. The dose of ML385 in rats was adopted from the published literature [40]. On Day 15, drug treatment commenced in rats that were selected based on the presence of established arthritis manifestations and significant paw edema. MTX and LIQ were dissolved in a 0.5% sodium carboxymethyl cellulose solution for oral gavage, while the control group and model group received the same volume of drug-free vehicle. ML385 was initially dissolved in 1% DMSO, then diluted to a working concentration using normal saline prior to intraperitoneal administration. The administration period lasted until Day 28, when 4 rats per group were randomly selected for terminal examinations and sample collection, after which all experimental rats were euthanized.

### 4.5. Paw-Swelling Degree and Arthritis Index

After the establishment of the CFA model, the general condition and behavioral changes of rats in all groups were monitored. From Day 3, the paw-swelling degree was determined using a plethysmometer (YLS-7B, Xingtaihuiyang Biotechnology, Xingtai, China). From Day 15, the arthritis index was assessed by grading the extent of joint erythema and swelling on a 0–4 scale for each hind paw, and scores were summed to yield a maximum of 8 points per animal [33]. All procedures were performed at 2-day intervals until the end of the administration period, with three repeated measurements.

### 4.6. Histopathology Analysis

Upon completion of the experiment, the hind ankle joint of the primary paw was carefully isolated, trimmed of surrounding soft tissue, and immersed in 4% paraformaldehyde for fixation. Following thorough decalcification with EDTA, the specimen was paraffin-embedded and serially sectioned in the sagittal plane before HE staining. HE sections were examined microscopically and histopathologically assessed using a 0–3 scale for three parameters: (A) periarticular soft tissue inflammation, (B) synovial hyperplasia, and (C) cartilage damage. A and B: 0, normal; 1, mild; 2, moderate; and 3, severe. C: 0, normal; 1, mild damage with granulation tissue; 2, moderate destruction with local defect and inflammatory infiltration; and 3, severe destruction with replacement by fibrous tissue. Cumulative scores ranged from 0 to 9 [41].

### 4.7. Micro-CT Analysis

Upon termination of the administration phase, a random sample of rats was taken from each group and anesthetized with pentobarbital sodium. The hind paw region was then scanned using a micro-CT scanner (PerkinElmer, Waltham, MA, USA). The scanned data were subjected to 3D reconstruction. Joint bone destruction was then assessed using a radiological scale graded from 0 to 3 according to the severity of bone damage from none (0) to mild (1), moderate (2), and severe (3) [42].

### 4.8. Immune Organ Index and Body Weight

The body mass of experimental rats was quantified using a digital balance from Day 3 onwards. Rats were euthanized using pentobarbital sodium at the conclusion of the experimental treatment. The spleen and thymus of rats were rapidly excised, and their fresh weights were immediately recorded. The immune organ index of rats was calculated using the formula:

Immune organ index = immune organ wet weight (mg)/body weight (g),

and served as indicators of CFA-induced lymphoid hyperplasia and the immunomodulatory effects of drug treatment.

### 4.9. Cell Culture and Drug Treatment

The MH7A (CVCL_0427) cells were obtained from Laibaiha Biotechnology (Shanghai, China). MH7A cells were cultured in DMEM enriched with 10% fetal bovine serum and were kept at 37 °C in a humidified atmosphere containing 5% CO_2_. TNF-α was employed to construct the RA-FLS cell model. MH7A cells were randomly allocated to control, model, graded concentrations of LIQ (10, 20, 40 μM) and ML385 (40 μM LIQ + 1.25 μM ML385) groups. The cellular concentration of ML385 was established based on prior reports in RA-FLS [43]. After seeding and pre-incubation, MH7A cells underwent pretreatment with the respective drugs for 1 h and were subsequently stimulated with 20 ng/mL TNF-α for 24 h prior to further experiments.

### 4.10. Cell Proliferation Assays

MTT assays were employed to investigate the effects of LIQ on the viability of MH7A cells, as well as its influence on the proliferation of MH7A cells stimulated by TNF-α. Initially, MH7A cells were inoculated into 96-well microplates and incubated with a concentration gradient of LIQ (1.25–320 μM) for 24 h, and then treated with MTT solution and cultured for a further 4 h. After removal of the supernatant, DMSO was used to dissolve the formazan precipitates, and the absorbance was recorded at 490 nm with a microplate reader (Molecular Devices, San Jose, CA, USA) to determine cell viability. Subsequently, newly seeded MH7A cells underwent pre-treatment with the respective drugs for 1 h, followed by TNF-α stimulation for 24 h. After MTT incubation and dissolution in DMSO, MH7A cell proliferation was assessed using identical methods and parameters.

### 4.11. Wound Healing Assays

After being plated in 6-well plates, the MH7A cells underwent incubation for 24 h before the initiation of the scratch. A sterile 200 μL pipette tip generated consistent vertical scratches in the cell monolayer; loosened cells were subsequently removed through gentle PBS washing. Each well was pre-treated with the respective drugs for 1 h, after which 20 ng/mL TNF-α was introduced to establish the inflammatory migration model. Imaging data were collected from the same microscopic region at 0 h (immediately after TNF-α stimulation) and 24 h via an inverted microscope; subsequently, wound area was analyzed using ImageJ 1.54g to determine the percentage of healed region.

### 4.12. Transwell Assays

Transwell assays were performed using 24-well plate inserts. Before cell seeding, the Transwell (8 μm) inserts were pre-coated with Matrigel (Solarbio, Beijing, China) to mimic the in vivo extracellular matrix environment. MH7A cells were reconstituted in 2% low-serum medium supplemented with the respective drugs and placed in the upper compartment, while the lower compartment was populated with a fully supplemented medium which included 10% fetal bovine serum and 20 ng/mL of TNF-α. After an incubation period of 24 h, the cells that did not migrate through the membrane were carefully removed, and the inserts were immersed in 4% paraformaldehyde prior to crystal violet staining. Microscopic examination was conducted to visualize and compare the distribution of migrated cells across treatment groups.

### 4.13. Intracellular ROS Measurement

ROS in MH7A cells were fluorescently labeled using the DCFH-DA probe. MH7A cells were seeded into confocal dishes. After pre-treatment with the drugs and exposure to 20 ng/mL TNF-α for 24 h, DCFH-DA was administered to the cells, which were subsequently maintained in the dark for 30 min. Following PBS washing, fluorescence signals were acquired via an LSM800 confocal microscope (Carl Zeiss, Jena, Thuringia, Germany).

### 4.14. Measurement of Cytokines IL-1β and IL-18

In vivo, the rat abdominal aorta served as the source for blood sampling, followed by centrifugation to isolate serum. In vitro, MH7A culture supernatants from each group were harvested and centrifuged. IL-1β and IL-18 levels were subsequently assessed via the respective ELISA kits in compliance with the manufacturers’ directions, and provided a comprehensive assessment of inflammatory status both in vivo and in vitro.

### 4.15. Immunofluorescence Staining

For immunofluorescence, MH7A cells seeded on confocal dishes were exposed to the respective drugs and challenged with 20 ng/mL TNF-α. The cells were then fixed in 4% paraformaldehyde for 30 min, permeabilized with 0.3% Triton X-100 for 15 min, and blocked with 5% bovine serum albumin for 1 h. Subsequently, the primary antibodies (Nrf2, p65, or NLRP3) were added and incubated with the cells overnight at 4 °C with a dilution of 1:100. Following the washing step, fluorescent secondary antibodies were added and incubated at ambient temperature with protection from light for 1 h. DAPI was subsequently applied for 10 min to stain the nuclei, after which images were acquired using a LSM800 confocal microscope.

### 4.16. Western Blotting

After drug treatment, MH7A cells and rat joint tissue samples were subjected to ultrasonication and lysis on ice in RIPA buffer that included a mixture of protease and phosphatase inhibitors. Subsequent to centrifugation, the supernatants were collected for total protein quantification via BCA kits (Beyotime, Shanghai, China). Sample proteins were loaded onto SDS-PAGE for electrophoresis and subsequently transferred to PVDF membranes (Millipore, Burlington, MA, USA). Membranes were incubated in Tris-buffered saline supplemented with 5% non-fat milk for 1 h at room temperature. Subsequently, the membranes were placed in a refrigerator at 4 °C and left to incubate overnight with primary antibodies specific to various target proteins, including Nrf2, HO-1, p65, p-p65, IκB-α, p-IκB-α, NLRP3, ASC, c-caspase-1 and GAPDH. All antibodies were used at a dilution of 1:1000. After washing, membranes were treated with horseradish peroxidase-tagged secondary antibodies under ambient conditions for 1 h. Immunoreactive bands were identified using an ImageQuant LAS 4000 imaging system (GE Healthcare Bio-Sciences, Pittsburgh, PA, USA) and semi-quantified by ImageJ software 1.54g.

### 4.17. Statistical Analysis

All experimental findings were presented as mean ± SD. The statistical analysis utilized GraphPad Prism 9.5 software (GraphPad Software Inc., San Diego, CA, USA). Intergroup differences were assessed through one-way analysis of variance (ANOVA) where appropriate. A *p*-value of less than 0.05 was considered statistically significant.

## 5. Conclusions

This research demonstrates that LIQ can alleviate RA severity and joint damage through modulation of inflammation and aberrant activation in FLS, which may be mechanistically attributed to regulating the Nrf2/NF-κB/NLRP3 pathway. In summary, these findings support the anti-RA potential of LIQ and may provide insights for future research on related natural products.

## Data Availability

The original contributions presented in this study are included in the article. Further inquiries can be directed to the corresponding authors.

## Abbreviations

ASC: apoptosis-associated speck-like protein containing a CARD; CFA: complete Freund’s adjuvant; DAMPs: damage-associated molecular patterns; DCFH-DA: 2′,7′-dichlorofluorescin diacetate; ELISA: enzyme-linked immunosorbent assay; FLS: fibroblast-like synoviocytes; HE: hematoxylin and eosin; HO-1: heme oxygenase-1; IL-18: interleukin-18; IL-1β: interleukin-1β; KEAP1: Kelch-like ECH-associated protein 1; LIQ: liquiritigenin; micro-CT: micro computed tomography; MTT: methyl thiazolyl tetrazolium bromide; MTX: methotrexate; NF-κB: nuclear factor-κB; NLRP3: NOD-like receptor pyrin domain-containing protein 3; Nrf2: nuclear factor erythroid 2-related factor 2; PAMPs: pathogen-associated molecular patterns; RA: rheumatoid arthritis; ROS: reactive oxygen species; TNF-α: tumor necrosis factor-α.
